# Development of a Maternal and Child mHealth Intervention With Aboriginal and Torres Strait Islander Mothers: Co-design Approach

**DOI:** 10.2196/33541

**Published:** 2022-07-08

**Authors:** Sarah Jane Perkes, Belinda Huntriss, Noelene Skinner, Bernise Leece, Rosie Dobson, Joerg Mattes, Kerry Hall, Billie Bonevski

**Affiliations:** 1 Flinders Health and Medical Research Institute College of Medicine and Public Health Flinders University Bedford Park, South Australia Australia; 2 School of Medicine and Public Health College of Health, Medicine and Wellbeing University of Newcastle Newcastle Australia; 3 National Institute for Health Innovation The University of Auckland Auckland New Zealand; 4 First Peoples Health Unit Griffith University Southport Australia

**Keywords:** mHealth, co-design, Aboriginal and Torres Strait Islander, mother, baby, young children, mobile phone

## Abstract

**Background:**

Despite their growing popularity, there are very few mobile health (mHealth) interventions for Aboriginal and Torres Strait Islander people that are culturally safe and evidence based. A co-design approach is considered a suitable methodology for developing health interventions with Aboriginal and Torres Strait Islander people.

**Objective:**

The aim of this study was to co-design an mHealth intervention to improve health knowledge, health behaviors, and access to health services for women caring for young Aboriginal and Torres Strait Islander children.

**Methods:**

Aboriginal researchers led engagement and recruitment with health services and participants in 3 Aboriginal and Torres Strait Islander communities in New South Wales, Australia. Focus groups and interviews were facilitated by researchers and an app developer to gather information on 3 predetermined themes: design characteristics, content modules, and features and functions. Findings from the co-design led to the development of an intervention prototype. Theories of health behavior change were used to underpin intervention components. Existing publicly available evidence-based information was used to develop content. Governance was provided by an Aboriginal advisory group.

**Results:**

In total, 31 mothers and 11 health professionals participated in 8 co-design focus groups and 12 interviews from June 2019 to September 2019. The 6 design characteristics identified as important were credibility, Aboriginal and Torres Strait Islander designs and cultural safety, family centeredness, supportive, simple to use, and confidential. The content includes 6 modules for women’s health: Smoke-free families, Safe drinking, Feeling good, Women’s business, Eating, and Exercising. The content also includes 6 modules for children’s health: Breathing well; Sleeping; Milestones; Feeding and eating; Vaccinations and medicines; and Ears, eyes, and teeth. In addition, 6 technology features and functions were identified: content feed, social connection, reminders, rewards, communication with health professionals, and use of videos.

**Conclusions:**

An mHealth intervention that included app, Facebook page, and SMS text messaging modalities was developed based on the co-design findings. The intervention incorporates health behavior change theory, evidence-based information, and the preferences of Aboriginal and Torres Strait Islander women and health professionals. A pilot study is now needed to assess the acceptability and feasibility of the intervention.

## Introduction

### Background

The health and well-being of Aboriginal and Torres Strait Islander people have been significantly affected by dispossession, interruption of culture, and intergenerational trauma since the colonization of Australia [1]. The ongoing impact has resulted in an unequal opportunity for good health. The life expectancy of Aboriginal and Torres Strait Islander women is 8 years less than that of non-Indigenous Australian women [2]. In the 2018-2019 National Aboriginal and Torres Strait Islander Health Survey, the majority of women aged ≥15 years were not meeting guidelines for physical activity, vegetable intake, or fruit intake; 36% reported that they smoked tobacco daily; and 35% reported that they experienced high or very high levels of psychological distress [3]. Infant mortality continues to be unacceptability high for Aboriginal and Torres Strait babies at 2.1 times the rate of non-Indigenous infants (6.3 and 3.1 per 1000 live births, respectively) [4]. Mothers and babies getting the best possible care and support for a good start to life is 1 of 12 health priorities of the National Aboriginal and Torres Strait Islander Health Plan 2013-2023 [1].

Aboriginal and Torres Strait Islander people make up 3.3% (798,400/24,193,939) of the Australian population [5] and include many distinct groups with their own language and culture. In total, 44% of Aboriginal and Torres Strait Islanders live in regional areas, 37% in cities, and 18% in remote or very remote areas [5]. Those living in regional and remote areas have less access to primary health care and overall poorer health [6]. Nationally, Aboriginal and Torres Strait Islander people have less access to the internet at home (75.3% compared with 85.8% of all Australians); there are significant differences based on location: 82.8% in cities, 73.2% in regional areas, 61.3% in remote areas, and 49.9% in very remote areas [7]. More than 1 in 3 (35%) Aboriginal and Torres Strait Islander people are mobile-only users compared with a national rate of 1 in 5 (19.9%); these figures are linked to socioeconomic factors [8]. Using only a mobile is likely to incur more costs for data, less capability, and less access to more sophisticated digital health information and tools [8]. It is of importance that mobile health (mHealth) interventions are developed with a goal to increase digital inclusion.

mHealth is the use of mobile technology to improve health. Functions include SMS text messaging, multimedia messaging service, voice, internet access, and software apps, which range in complexity. mHealth is used for a variety of purposes, including health education, health behavior change, sensors and point-of-care diagnostics, registries and vital-event tracking, and data collection [9]. mHealth is being used increasingly for health promotion because of its reach, with >7 billion mobile phone subscriptions globally [10]; the Be He@lthy, Be Mobile initiative by the World Health Organization has reached >3.5 million people [11]. There are limited recent national figures on smartphone ownership among Aboriginal and Torres Strait Islander people, although available data indicate that ownership is high; a survey with 400 Aboriginal and Torres Strait Islander people in 2014 reported that 70% of Aboriginal and Torres Strait Islander people owned a smartphone and 69% used Facebook compared with 66% and 40% respectively for non-Indigenous Australians [12]. The top reason for using a mobile phone in this group was to send SMS text messages [12].

Studies focused on Aboriginal and Torres Strait Islander people using SMS text messaging to improve health show high acceptability of the modality [13-15]. SMS text messaging has the advantage of being accessible on all mobile phones and not requiring access to a data service. There are few technical barriers to SMS text messaging and high acceptability of the modality among new mothers [16,17]. In a metareview (23 systematic reviews, 371 studies, and 79,665 participants) on the impact of mHealth on a range of outcomes, including clinical outcomes, adherence to treatment and care, health behavior change, disease management, and attendance rates, SMS text messaging was the most frequently examined function and reported to be the most successful overall [18]. SMS text messaging seems to be particularly effective at increasing smoking cessation rates (in adult smokers from mostly high-income countries) [19]. The evidence for SMS text messaging helping to improve nutrition and physical activity is not as strong; however, SMS text messaging used in conjunction with other mHealth functionality has shown significant positive effects for healthy eating [18].

Health apps continue to be popular, although the evidence suggests that apps have limited effectiveness on changing health behaviors [18,20-22]. Some studies have found that apps can be effective at changing behavior among some clinical groups [18], although overall there is limited evidence to date. Of the few trials focused on Indigenous populations, app use has been reported to be low [23,24]. A recent pilot randomized controlled trial of a smoking cessation app with 49 Aboriginal people in Australia reported low to moderate level of app use, and at 6-month follow-up, only 1 participant was abstinent [24]. The authors concluded that although there was broad acceptability for the app, mHealth interventions should be designed with functions that are commonly used, including social media platforms [24]. A co-designed mHealth app developed in New Zealand with Māori and Pacific Islander people was tested in a cluster randomized controlled trial in 2019 (n=1451) [23]. Adherence to health-related–behavior guidelines increased at 12 weeks in both groups, with no difference between the groups. Engagement with the app overall was low, although those who did engage with the app as it was designed saw greater benefit. The co-design approach was reported to have drawn a very positive response from the community, as was reflected in the high participation and follow-up rates [23].

Social media is a form of mHealth, with potential to support health. The Aboriginal and Torres Strait Islander health sector was an early adopter of social media networks to promote health [25,26]. Social media campaigns on COVID-19 by Aboriginal and Torres Strait Islander health organizations is a recent example [27]. A recent Cochrane review on behavioral interventions delivered through social media for health behavior change, health outcomes, and health equity (88 studies; n=871,378) reported varied effects; overall, social media was found to improve physical activity, weight loss, and general well-being, and small to no effects were found for other outcomes [28]. No studies focusing on Aboriginal and Torres Strait Islander people were included in the review.

### Objectives

In response to the limited mHealth interventions available for Aboriginal and Torres Strait Islander women and children, we aimed to co-design a prototype focused on the needs and ideas of Aboriginal and Torres Strait Islander mothers. Co-design is a partnership approach where end users are actively involved from conception to dissemination [29]. Using co-design methodologies is one of the guiding principles of the Aboriginal Health and Medical Research Council of New South Wales (NSW) Ethical Guidelines for conducting health research with Aboriginal people [30]. In this paper, we describe the co-design processes and findings, as well as provide a description of the mHealth prototype.

## Methods

### Study Design

In total, 8 focus groups and 12 interviews were conducted from June 2019 to September 2019. Surveys were used to collect demographics at the start of focus groups and interviews. An Aboriginal advisory group that included Aboriginal team members who were also members of the participating communities met quarterly to oversee design, implementation, analysis, and reporting. An expert mHealth research group was consulted for opinion on research and intervention design.

### Ethics Approval

Human research ethics approval was received from the Aboriginal Health and Medical Research Council (1485/19) and the University of Newcastle (H-2019-00760).

### Co-design Framework

A co-design framework for an mHealth intervention with Māori and Pacific communities in New Zealand [29] based on work by Bratteteig et al [31] was used to guide the methods used in this study. Co-design is a coherent methodology with a range of tools and techniques used to favor the preferences of end users [31]. The co-design methods used included focus group and interview discussions, card sorting, storyboarding, design activities, survey, guidance from expert groups, and an iterative design phase with the research team.

### Setting

Focus groups and interviews were held at 3 regional NSW locations: Newcastle, Coffs Harbour, and Inverell. In total, 5 Aboriginal organizations (including 3 Aboriginal health services, an Aboriginal preschool, and an Aboriginal corporation) and 3 NSW Health sites participated. Venues for focus groups and interviews were decided in consultation with participants.

### Participants

Women aged ≥16 years who were either mothers or primary carers of an Aboriginal or Torres Strait Islander child aged 0 to 5 years or were pregnant (≥30 weeks gestation), owned or regularly used a smartphone, and had accessed a participating service (Aboriginal health service or NSW Health service) were eligible to participate. Health professionals at participating services who worked with women or children were eligible.

### Procedures

Convenience sampling was used to recruit participants. Aboriginal researchers (BH, NS, and BL) who worked within the participating communities used their personal networks. In addition, participants were asked if they would like to recommend a friend or family member to the study. Potential participants were screened for eligibility when they contacted the researcher on the telephone. The researcher explained the study and gained informed consent over the telephone initially and again in person before the start of the focus group or interview. Participants were reimbursed with a shopping voucher worth Aus $30 (US $21.6) for attending focus groups and interviews and provided with refreshments. Health professionals were recruited using a snowball methodology through the participating services. Health professionals were not reimbursed.

Mothers and health professionals participated in separate focus groups and interviews. Focus groups and interviews were cofacilitated by a combination of Aboriginal researchers (NS and BH), a PhD student (SJP), and an app developer. Interviews and focus groups were 20 to 90 minutes in length. The number of participants in focus groups ranged from 2 to 6. Focus groups and interviews were recorded and transcribed, and field notes were taken.

### Measures

Different surveys and discussion guides were used with mothers and health professionals. Discussions and activities were used to identify (1) design characteristics, (2) content modules, and (3) features and functions.

#### Mothers

##### Survey

The survey comprised 16 items, including demographic, cultural, and socioeconomic items. The items were selected from a previous study [32], with all items having been tested with Aboriginal and Torres Strait Islander mothers previously.

##### Discussion Guide

In all focus groups and interviews with mothers, 3 main questions were asked. Follow-up questions were asked depending on responses. Additional questions about mobile phone use to inform features and functions were asked in focus groups cofacilitated by the app developer. The three main questions were as follows:

How would an mHealth intervention designed for healthy living for Aboriginal and Torres Strait Islander people differ from other mHealth interventions?

Are you more interested in mHealth for your own health or your child’s health? What topics and features interest you?

What do you think stops or prevents some women from accessing health information and services for themselves and their children?

##### Activities

Card-sorting activities were used to identify current mobile phone use (functions used, frequency of use, and reasons for use). Storyboarding activity was used to elicit creative descriptions of the mHealth intervention using drawings and words on what the intervention should include. Design activity was used to gain feedback on potential designs.

#### Health Professionals

##### Survey

The survey comprised 5 items related to demographic and professional practice characteristics.

##### Discussion Guide

In all focus groups and interviews with health professionals, 3 main questions were asked. Additional follow-up questions were asked depending on the response. The three main discussion questions were as follows:

1. What do you think are the most important health and well-being topics to include for Aboriginal or Torres Strait Islander women, children, and family?
2. What are the barriers for Aboriginal or Torres Strait Islander families to having good health?
3. What types of mobile technology do you think could support Aboriginal or Torres Strait Islander women’s and children’s health?

#### Co-design Analysis

A generalized thematic analysis was completed. An Aboriginal researcher (BH) and a PhD student (SJP) independently coded themes. NVivo software (version 12.0; QSR International) was used to complete independent coding and comparison by the 2 coders. In total, 3 predetermined codes were used based on a similar co-design study [29]. These codes included (1) design characteristics, (2) content modules, and (3) features and functions. The coders met to agree on subcodes and definitions. Survey findings are presented using descriptive statistics.

#### Intervention Development

The findings from the co-design stage were subsequently used to develop a prototype intervention incorporating an app, SMS text messaging, social media, and videos. The intervention development was an iterative process, with meetings held among the team members to decide the final features and functionalities. Not all ideas could be adopted because of various reasons, such as time, funding, and technology constraints. We used a combination of building new functions (app) and using existing functions (Facebook page and SMS text messaging).

The intervention was grounded in behavior change theory. The Health Belief Model was used to underpin the app portion of the intervention. The Health Belief Model is considered to be well suited to mHealth interventions with use of the *cue to action* component [33]. The basic constructs are perceived threat of illness, perceived benefits of health behavior change, perceived barriers to change, cues to action, and self-efficacy [34]. Behavior change techniques were used to formulate SMS text messages. The SMS text messages were coded for behavior change techniques by 2 coders (Sam McCrabb and SJP) using behavior change technique taxonomy (version 1) [35] and the process outlined by Michie et al [36]. Of the 2 coders, 1 was experienced in coding behavior change techniques (Sam McCrabb) and the other was a PhD student (SJP). Disagreements were resolved through discussion and key messages adapted to include further effective behavior change techniques.

Key messages were developed on health topics identified from the focus groups and interviews. Content was formulated from publicly available evidence-based health resources. Key messages were adapted to SMS text messages, small pieces of written information for the app, and Facebook posts.

The prototype intervention included an app, videos, Facebook page, and SMS text messaging (Textbox 1).

**Components of the prototype intervention.**

**App**

- A web-based prototype app was developed. Rapid iterative cycles between the app developer and research team were used to refine the design. An Aboriginal graphic designer developed graphics for each module and logo.

**Videos**

- A total of 12 short videos were captured on a Canon camera. All presenters were health professionals from participating sites or contacts of the research team. Short scripts were provided to health professionals based on key messages. Staff were encouraged to use their own knowledge and expertise on each topic. Videos were filmed by a videographer and professionally edited. Captions were completed by Rev, and voiceovers were completed by 2 Aboriginal researchers (BH and NS). The videos ranged from 112 to 300 seconds in length. Vimeo was used as the platform to host the videos.

**Facebook page**

- A Facebook group was developed and administrated by 2 Aboriginal researchers (BH and NS). Both researchers were regular Facebook users and had significant networks and knowledge of Aboriginal and Torres Strait Islander organizations, events, and health services. Key messages were predeveloped in text and video format. Other content shared was decided by the administrators, including sharing posts from their personal accounts if they were suited to the broad aim of the intervention.

**SMS text messaging**

- SMS text messages were developed based on the processes described by Abroms et al [37]. Steps include choosing a behavior change goal, choosing communication objectives and behavioral techniques, designing a framework, and writing an SMS text message library [37]. SMS text messages were written to allow tailoring using the mother’s and child’s names, child’s age, and topic interest of the mother. Tailoring SMS text messages around the timing of key behaviors, such as after a baby is born, can improve saliency and likelihood of behavior change [38]. SMS text messages were written by an Aboriginal researcher (BH) and a PhD student (SJP). A web-hosted SMS text messaging server (SMS Express) will be used to send all SMS text messages.

## Results

### Overview

A total of 42 participants were recruited to the study: 31 mothers and 11 health professionals. Demographics and cultural characteristics of mothers are presented in Table 1, and demographics of health professionals in Table 2.

**Table 1:** Demographic and cultural characteristics of mothers (N=31).

Characteristics					Values
Age (years), mean (SD; range)					31.17 (7.69; 19-50)
**Indigenous status, n (%)**					
	Aboriginal			21 (68)	
	Torres Strait Islander			2 (7)	
	Nonidentified			7 (23)	
	Did not answer			1 (3)	
**Identified with an Indigenous community, n (%)**					
	Yes			25 (81)	
	No			1 (3)	
	Unknown			4 (13)	
	Did not answer			1 (3)	
Maintain cultural connections at home, yes, n (%)					25 (81)
**Ways of connecting to culture, n (%)**					
	Music or dance			19 (61)	
	Storytelling			19 (61)	
	Indigenous television			18 (58)	
	Art			15 (48)	
	Food			14 (45)	
	Indigenous internet sites			10 (32)	
	Indigenous newspapers			7 (23)	
	Traditional medicine			6 (19)	
	Indigenous radio			5 (16)	
	Other			1 (3)	
**Family members from Stolen Generations^a^, n (%)**					
	Yes			6 (19)	
	No			12 (39)	
	Unknown			13 (42)	
**Education of mother, n (%)**					
	Did not finish high school			6 (19)	
	High school			6 (19)	
	Certificate			10 (32)	
	Diploma			2 (7)	
	Bachelor’s degree			4 (13)	
	Postgraduate degree			1 (3)	
	Did not answer			2 (7)	
Currently pregnant, yes, n (%)					1 (3)
Partner, yes, n (%)					16 (52)
Number of people living in household, mean (SD; range)					4 (1.31; 2-7)
Number of children (aged <18 years) living in household, mean (SD; range)					2.39 (1.41; 1-5)
**Smoking status of mother, n (%)**					
			Nonsmoker	21 (68)	
			Yes, daily	5 (16)	
			Yes, at least once a week	2 (7)	
			Yes, less often than once a week	1 (3)	
			Did not answer	2 (7)	
Number of cigarettes smoked per day (on the days smoking), mean (SD; range)					8.5 (3.21; 4-12)
**Number of smokers in household, n (%)**					
		0			14 (45)
		1			10 (32)
		2 to 3			4 (13)
		>3			1 (3)
Child exposure to indoor tobacco smoke, yes, n (%)					1 (3)
Child exposure to outdoor tobacco smoke, yes, n (%)					15 (48)
Child exposure to tobacco smoke in the car, yes, n (%)					0 (0)

^a^The Stolen Generations refers to a period in Australia’s history when Aboriginal children were removed from their families through government policies. This happened during the period from the mid-1800s to the 1970s [39].

**Table 2:** Demographics of health professionals (N=11).

Characteristics		Values
**Health service type, n (%)**		
	Aboriginal medical service	6 (55)
	NSW^a^ Health service	5 (45)
	Sex: female	11 (100)
**Indigenous status, n (%)**		
	Aboriginal	4 (36)
	Torres Strait Islander	0 (0)
	Nonidentified	7 (64)
**Role at health service, n (%)**		
	Registered nurse	7 (64)
	Aboriginal health worker	3 (27)
	Senior family health practitioner	1 (9)
Number of years at service, mean (SD; range)		12 (8.7; 3-32)

^a^NSW: New South Wales.

### Design Characteristics

We identified six main design characteristics: (1) credibility, (2) Aboriginal and Torres Strait Islander designs and cultural safety, (3) family centeredness, (4) supportive, (5) simple to use, and (6) confidential.

#### Credibility

Mothers talked about the difficulty of finding information on the web that was evidence based. Most of the mothers said that they used Google to find real-time health information for themselves and for their children: “Literally, I Google everything.” Many of the mothers said that it can be difficult to know which websites are most up to date and accurate and that it is difficult to find information: “The biggest thing I find on Google, you get everything. You don’t get the ones that are reputable.” Another mother said, “I'm finding you’re having to like scroll, scroll, and scroll to try and find that information.” Mothers said that they want current health information from reputable health professionals and organizations, including “useful websites links.” Health professionals talked about the importance of credible health information to improve health literacy: “I think lack of knowledge that they are so sick. Recognizing the signs of illness that can lead to them being really, really [sick].” This highlighted why it is important that all content included in the prototype intervention be sourced from credible evidence-based health resources and broken down into palatable small chunks with links to further information.

#### Aboriginal and Torres Strait Islander Designs and Cultural Safety

Most of the mothers said that Aboriginal designs, language, and representation were important for engagement. A mother said, “I think if it had Aboriginal designs that would be really good because if I download an app and it doesn’t have the look, like being culturally aware [I don’t use it].” Another mother said, “Don’t make it black and white, it’s got to be like colorful.” A mother spoke about the intervention needing Aboriginal representation in images and videos: “If it’s going to be an Aboriginal app, I think you have to have Aboriginal people.” Another mother discussed using an app for quitting smoking that was not representative of Aboriginal people: “It was easy to use, but I couldn’t relate to it...didn’t seem like it was aimed at Blackfellas even though we thought it was.”

It was evident from the mothers’ experiences of racism that the intervention needed to be centered in culturally safety. Some mothers talked about feeling fearful and judged when seeking health care. A mother said, “Being an Aboriginal mum especially, I was just worried about DoCS [Department of Child Services]. Like whether they could see if I was handling having two children on top of my own family breakdown. Like my mum’s kids are in DoCS. So that’s what my biggest fear was.” Other mothers expressed feeling judged about certain health behaviors and topics, and a mother said, “The biggest thing is why people do hide it [smoking], because they don’t want to be judged. They don’t want to hear all that stuff.”

To center cultural safety in the intervention, all aspects of the intervention were codeveloped by Aboriginal people: the research was governed by an Aboriginal advisory board and coled by an Aboriginal academic (KH); 4 of the 8 members of the research team are Aboriginal; an Aboriginal graphic designer designed the module icons and logo; Aboriginal researchers were administrators of the Facebook page and shared cultural links, events, activities, affirmations, and images; an Aboriginal videographer filmed all the videos; Aboriginal health professionals presented in the videos; an acknowledgment of Country and a *welcome* message by an Aboriginal researcher was placed on the main page; and all content was cowritten by Aboriginal researchers.

#### Family Centeredness

It was decided unanimously that the intervention should include content for both mother and child. A mother said, “Is this just for children’s health? Because I feel like it should incorporate the mother’s health too.” The mothers asked for information on “things to do with our kids,” and “stuff for us women too. Pap smears and stuff like that.” Many of the mothers and health professionals suggested that the intervention needed to encompass the entire family, including the extended family. A health professional said, “Put the main focus on the child and then how their [family] health affects the baby’s health,” and a mother said, “I think a family app would be really good. Like, I know my husband, he’s never been around babies.” Some participants talked about how other family members help bring up children: “It’s nothing to see an aunt bringing up a child, or a grandparent or a sister” [health professional]. Family centeredness in the intervention was therefore conveyed through messaging that families are the most important role models for jarjums (an Aboriginal word meaning children) across modules and functions. Links to websites, events, and health information for partners and other family members were included.

#### Supportive

Most of the mothers and health professionals indicated that it was important that the intervention promoted positive self-esteem and well-being of mothers. A health professional said that the intervention should give new mothers “understanding [of] how tired you are going to be, and it’s okay, ask for help, everyone feels like that but you’re not failing or not doing something wrong.” A mother suggested that we include “some sanity sayings or something like that, or some little sage advice from mums that have been there, done that before, that’d be really helpful,” and another mother said that the intervention could be “like a reassurance type thing.” Mothers and health professionals recognized that motherhood can be “totally exhausting” [health professional] and challenging at times. A mother described the initial period after coming home from hospital: “I didn't know what to do with him. What do I do with this kid? I was lost.” To create an intervention that was supportive of motherhood and of Aboriginal and Torres Strait Islander women, positive and affirming messages were posted on Facebook, sent through SMS text messages, and included in the app. Links on where to seek help for mental health concerns were included.

#### Simple to Use

Mothers and health professionals recommended that the intervention be intuitive, use simple language, and have few technical barriers. Some of the mothers talked about trying to use other health apps; however, they were unable to do so because of technological challenges. For example, a mother said, “It was just too hard to log in and get started so I gave up or just called someone.” Many of the mothers and health professionals emphasized that the language used in the intervention needed to be nonjargon. A mother said, “Don’t put it in a textbook. Because I’m telling you, if my family member downloaded that and it was a textbook way, they would be like—No.” Another mother said that the content should be “just little pieces of information...then links to the bigger pieces.” We aimed for simple, intuitive app design and used other mobile functions commonly used by mothers (Facebook and SMS text messaging). To ensure that the intervention was simple and easy to use, health information was presented in short key messages with links to websites for further information. All key messages were written to be at an 8th grade reading level using the Flesch-Kincaid Grade Level Test as recommended by Abroms et al [37].

#### Confidential

Mothers and health professionals talked about the importance of confidentiality. Health professionals focused on confidentiality in the health care setting and the complexities for some staff regarding knowing patient health details. A health professional said, “There are big things surrounding our health services confidentiality. People don’t know or want to know what other people’s business is.” Some of the mothers spoke about confidentiality; regarding being anonymous when communicating with other mothers or health professionals in a hypothetical mHealth intervention, a mother said, “Oh God, yeah. I’d ask an anonymous person on a phone. Rather than ask the doctor face to face.” Other mothers were happy to not be anonymous: “It wouldn’t bother me having my name because it would just be, this is my experience, and it is what it is. But I would understand if some women didn’t.” To ensure that women can choose to remain anonymous and keep their information confidential, the intervention design meant that no personal data were collected in any part of the intervention, other than a mobile number for the SMS text messaging component. Joining the Facebook group is an optional part of the intervention.

### Content Modules

Most of the mothers and health professionals suggested that the intervention needed to cover a wide range of health topics for both the mother and child. Health topics identified in the data included *pains after birth*, *breastfeeding*, *normal speech for toddlers*, *signs of autism*, *earaches*, *behavior*, *rashes*, *high temperatures*, and *coughs*. Similar topics were grouped by the research team and combined into 6 key content modules for women’s health and 6 key content modules for children’s health. For example, *birth*, *reproductive health*, *urinary leaking*, and *pap smears* became *Women’s business*. All health topics captured in the interviews and focus groups were included in the intervention within a module on the app, SMS text messages, or through Facebook posts. Health modules for women included Smoke-free families, Safe drinking, Feeling good, Women’s business, Eating, and Exercising. Health modules for children were Breathing well; Sleeping; Milestones; Feeding and eating; Vaccinations and medicines; and Ears, eyes, and teeth.

### Features and Functions

We identified eight features and functions: (1) content feed, (2) social connection, (3) diary and storage of health information, (4) local context, (5) reminders, (6) rewards, (7) talk with health professionals, and (8) use of videos.

#### Content Feed

A content feed was chosen to be a feature of the intervention based on the mothers’ current mobile phone use. During the card-sorting activity, most of the mothers reported scrolling the content feed on Facebook numerous times per day. Of the 13 women who were asked how many hours per day they used Facebook, 12 (92%) reported using it >4 hours per day. When asked what kept them going back to Facebook, a mother responded, “The content keeps changing.” Mothers frequently talked about watching photo and video stories that were uplifting, funny, or motivating on Facebook. They talked about using Instagram and Snapchat, too, although less frequently. The intervention was therefore designed to include a Facebook page with daily posts covering a variety of health content.

#### Social Connection

Mothers talked about the social connection and learning from other women when becoming a mother, including from their “mum,” “mother-in-law,” and “girlfriends.” The importance of positive relationships when first becoming a mother was well recognized by health professionals as well as mothers. It was acknowledged by many of the mothers that some new mothers “don’t have a big support network.*”* A mother described mothers at playgroup being “more like a family to each other.” Some of the mothers said that connecting to other mothers would be helpful because they may be going through the same situation or challenge: “Yeah [I would like to chat with mums in the intervention] because they might have experienced something that I’m starting to experience.” Some of the mothers talked about the possibility of meeting up with mothers outside of the intervention: “It’s hard to meet people...[could there be] like a mums and bubs [babies] thing [as part of the intervention],” and another mother said, “Say, if I needed to ask them a question or something that I wouldn’t want to write on Facebook [I would like to meet up with them in person].” Another mother identified that connection is important for mental health: “When they [new mothers] don’t have anybody, depression kicks in.” The Facebook page was designed to make it easy for mothers to connect and share stories and ideas. Discussion points were created to be posted on the Facebook page to facilitate discussion; for example, “Tell us how you engage your jarjums in cooking or take a pic or video of your deadly (great or excellent) li’l chef in the kitchen.”

#### Diary and Storage of Health Information:

A feature that enabled users to store specific information about a child’s health received mixed responses. Some of the mothers thought that having their child’s health information on hand would be of practical benefit when attending medical appointments: “Like a diary section...I found, when [my child] was sick I started recording when I gave the medication, those sorts of things. That’d be good to have an app when you go into the hospital, you go, this is his recordings.” Another mother said, “So they [health professionals] could just add in medication, add in reports...it’d be good because like [the health service] is only open during the week. Usually, like on the weekend, I’d have to go up to the hospital...So it would be good if there was information like after the visit. Because you don’t always take everything in. It goes right over your head.” Other mothers and health professionals thought there would be confidentiality concerns. Because of the confidentiality concerns raised in the co-design process, a diary feature was not included, although it may be considered as an optional feature in future iterations.

#### Local Context

Many of the mothers and health professionals spoke about the uniqueness of their community and said that the intervention needed to be relevant to each community, including language and environment (eg, coastal and desert), as well as health services and other resources. A mother suggested, “You could put in your postcode, location, or area or something and then it could be localized,” and a health professional said, “The contact numbers, if they can’t get into emergency, the [local] health line numbers where they can get a bit of advice would be handy on there as well.” The intervention included phone numbers of local health services for each community in the app, and Facebook posts were designed promoting local health services, events, organizations, and languages.

#### Reminders

Many of the mothers talked about the usefulness of SMS text messaging reminders from their health services for appointments, and they said that reminders for other areas of health care would be useful too. A mother said, “I would probably like all of them [milestone reminders]. I’d like the whole lot, make sure I’m not missing anything.” Another mother said, “If someone notified me on this app that I’m due for a [pap smear] or something like that, I would like being reminded of things like that.” Most of the mothers said that they would prefer reminders through SMS text messages rather than a push notification from an app because they could go back to the message and reread it. For the intervention, SMS text messages were developed covering a range of reminders, including vaccinations, developmental milestones, check-ups, smoking quit date, exercise, and eating well. Reminders about local health initiatives and events were also created for posting on the Facebook page.

#### Rewards

The mothers talked about rewards and incentives from health programs and services increasing their motivation. They talked about material rewards such as “shirts,” “caps,” and “supermarket vouchers,” as well as social rewards, including “comments” and “likes” on social media and “clapping” and “cheers” on health apps*.* The mothers who were asked about receiving rewards for a variety of health behaviors were unanimous in their opinion that rewards were enjoyable and motivating*.* In the intervention, weekly competitions were created for posting on the Facebook page involving mothers sharing a picture of a health activity; for example, active play or exercising with their children. Prize draws were also incorporated into the intervention for those who participated in the competitions.

#### Talk With Health Professionals

Some of the mothers suggested that being able to communicate with health professionals using SMS text messages or a live chat function would be beneficial. Some of the mothers said that this function would be useful to confirm whether they required face-to-face health care and for reassurance. A mother said, “Sometimes you don’t know if you should go up there [health service] or not, so you could kind of message and say, ‘Hey, this is what’s happening...is it worth coming up or is it just a viral thing going around?’” Another mother said, “I know a lot of women are just like, ‘What do I do?’ So just having that reassurance I suppose online.” Another mother suggested that it would be helpful to be able to ask health questions anonymously: *“*The option to be anonymous or not known by people [health professionals] would be handy I guess for more embarrassing health concerns.” Mothers living in rural areas mentioned being anonymous more often in the discussions. Although it was suggested, facilitating a chat with health professionals directly was out of the scope of the current prototype because of cost and resources. Telephone numbers for national, state, and local health services were listed in the app to enable users to connect with health professionals, if needed, regarding the questions they might have.

#### Use of Videos

Most of the mothers reported during the card-sorting activity that they frequently watched short videos on social media and YouTube. A number of mothers and health professionals advised us that videos and images may be more accessible and preferable for some mothers. A health professional said, “Videos, everyone can watch a video and understand.” Therefore, a video for each health module was developed for the intervention. Each video was stored in the app and added to the Facebook page. Additional health videos from external sources were also able to be shared on the Facebook page.

### Final Prototype

The final mHealth intervention, named Growin’ Up Healthy Jarjums, aimed to improve health knowledge and health behaviors, along with providing access to health services. The intervention comprises 3 delivery modalities: app, SMS text messaging, and Facebook page.

#### App

The app is a central place for users to access all content. The app is primarily for the user who wants in-depth information and has the necessary digital device, internet connection, and literacy skills to access it. It is designed to allow the user to navigate to the topic of interest; for example, *exercise,* where they will find small amounts of written information, videos, links to websites, and useful contacts. The user may choose to access any topic, in any order, and consume as much information as they like.

**Figure 1:**
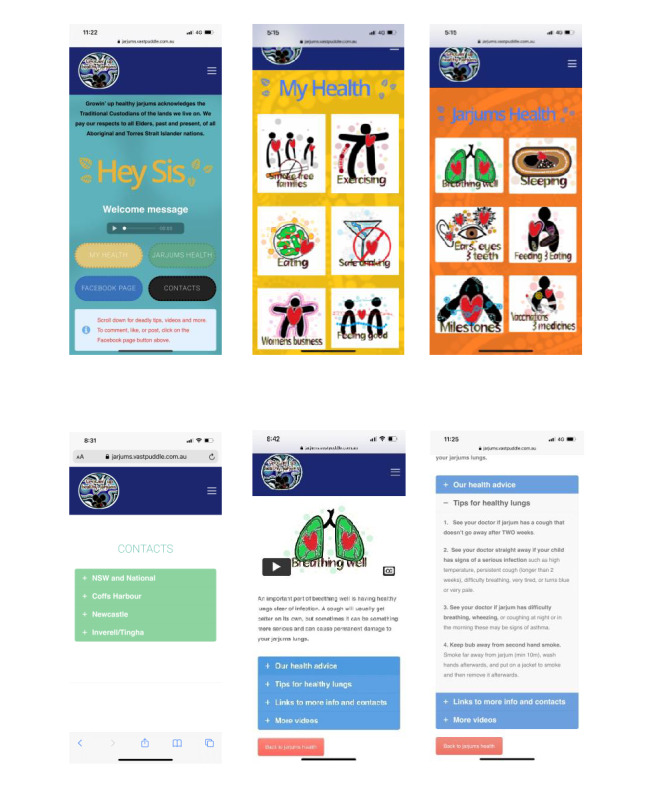
Examples of Growin’ Up Healthy Jarjums app screens: (top, from left) home, women’s menu, and children’s menu; (above, from left) contacts, Breathing well, and Our health advice (accessed from Breathing well).

The app has four menu screens: (1) home screen, (2) women’s health, (3) children’s health, and (4) contacts (Figure 1). The home screen includes four buttons: (1) *My Health*, (2) *Jarjums Health*, (3) *Facebook Page*, and (4) *Contacts*. The user may click on a button to move to the next screen or scroll down to access the embedded Facebook content feed. The embedded Facebook content feed allows the user to remain in the app and read the posts, but to comment or *like* a post, the user needs to access the Growin’ Up Healthy Jarjums Facebook page. An acknowledgment of Country and a spoken welcome message are also included on the home screen. The women’s health (*My Health*) menu page includes six buttons, one for each of the women’s health modules: (1) Smoke-free families, (2) Safe drinking, (3) Feeling good, (4) Women’s business, (5) Eating, and (6) Exercising. The *Jarjum’s Health* menu page has the same layout, including six buttons for the children’s health modules: (1) Breathing well; (2) Sleeping; (3) Milestones; (4) Feeding and eating; (5) Vaccinations and medicines; and (6) Ears, eyes, and teeth. Each module, for example, *Breathing well*, includes (1) *Key messages* incorporating perceived threat of illness and benefits of changing health behavior; (2) *Tips* to address barriers to change through reassurance and credible advice; (3) cues to action; for example, “Each time jarjum sees a nurse or GP ask them to have a quick look in bub’s ears to check if there is any infection”; and (4) links to further information, including skills and activities; for example, exercises and healthy recipes to support self-efficacy. The information is presented using small chunks of written information and videos using the same layout in each module.

#### SMS Text Messaging

Alongside the app, the prototype included an SMS text messaging library comprising 112 SMS text messages (Table 3). The SMS text messaging component allows users access to health information regardless of mobile phone type, Wi-Fi access, or digital literacy. The SMS text messages covered the content topics identified by the participants. The SMS text messaging portion of the program is 1-way (unidirectional), other than 3 SMS text messages developed for users who indicate that they want to quit smoking when registering for the program. In total, 23 behavior change techniques from 15 behavior change clusters were incorporated in the SMS text messages (Multimedia Appendix 1).

**Table 3:** Example SMS text messages developed for the Growin’ Up Healthy Jarjums modules.

Module		Example SMS text message
**Women’s health**		
	Smoke-free families	Text4jarjum: Giving up the smokes is the best thing you can do for your health. Be a role model and be smoke free. Get support from Quitline 13 78 48 or a doctor and quit for good!
	Safe drinking	Text4jarjum: While under the influence of alcohol, people can make less safe decisions about their jarjums. Check out 'Safe drinking' for tips to set limits.
	Feeling good	Text4jarjum: You’re probably not getting much sleep right now. Try to make time for yourself, ask for support from family & friends, and nap when bub does. If you feel that you are not coping, talk to your doctor or midwife. There is help.
	Women’s business	Text4jarjum: Be kind to yourself. Your body has gone through some big changes during and after birth. It will take time to bounce back. Whether you had a caesarean or vaginal birth, both may require rest & time for recovery. Here’s what to expect after birth.
	Eating	Text4jarjum: The Australian Breastfeeding Association has some useful tips on nutritional needs for breastfeeding mums.
	Exercising	Text4jarjum: Any amount of movement is good for you. Start by doing a little, and gradually build up. You could start with a walk around the block a few times a week and then gradually increase.
**Children’s health**		
	Breathing well	Text4jarjum: A cough is often caused by a cold. Usually, a cough gets better on its own and is not serious, but if your child has a cough that doesn’t go away after TWO weeks, or if you are concerned sooner – see your doctor or child health nurse.
	Sleeping	Text4jarjum: A routine that includes relaxing time like bath, book, a gentle song before bed and a regular bedtime each night can help your child settle better.
	Milestones	Text4jarjum: Playgroups, day care and pre-school are great places for jarjums to play and develop. Contact your AMS (Aboriginal Medical Service) or health nurse and find out what’s on.
	Ears, eyes, and teeth	Text4jarjum: Ear infections are really common and can cause long term hearing loss if not treated. Often there are no signs. Ask your doctor to have quick look in [insert child name] ears each visit to make sure there is no infection.
	Vaccinations and medicines	Text4jarjum: Immunising [insert child name] is a safe and easy way to keep jarjums healthy and prevent disease. To check that [insert child name] is up to date with immunisations click here.
	Feeding and eating	Text4jarjum: It’s recommended you breastfeed exclusively until [insert child name] starts solid foods at around 6 months of age. Keep breastfeeding until at least 12 months and beyond.

#### Facebook Page

**Figure 2:**
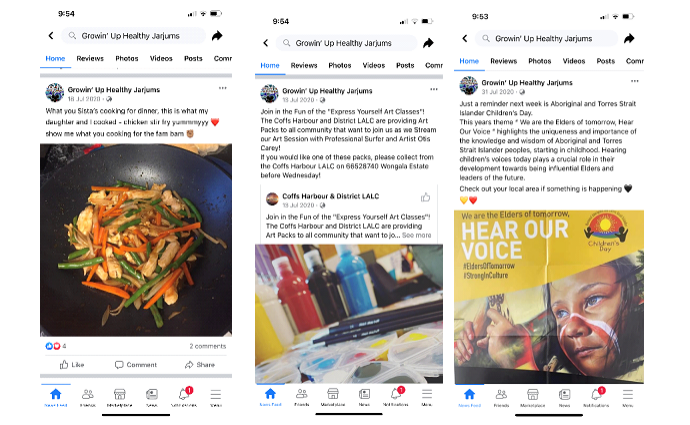
Examples of the content feed shared on the Growin’ Up Healthy Jarjums Facebook page.

The final modality included in the prototype was the Facebook page. The purpose of the Facebook page was to create community and connection, allow 2-way communication, and use a platform that is highly popular among users. Daily content was designed to be added to the Facebook page, including (1) links to reliable health websites, (2) activities for families, (3) weekly competitions, (4) key messages (written and video), (5) events in the community, and (6) supportive affirmative posts. The page was administrated by 2 Aboriginal team members (NS and BH), who shared posts relevant to their community and region. The Facebook page was embedded into the main screen of the app; it could also be accessed through Facebook. Examples of posts are presented in Figure 2.

## Discussion

### Principal Findings

We codeveloped a prototype mHealth intervention focused on the knowledge of mothers of young Aboriginal and Torres Strait Islander children. The aim of the intervention was to improve health knowledge, health behaviors, and access to health services. The final prototype incorporates 3 modalities—app, SMS text messaging, and Facebook page—and includes a range of health topics. In addition, it is centered on being supportive of mothers and culturally safe.

The modality choices were based on a few factors: (1) early discussions with mothers and health services about the need for an app that is culturally relevant and safe, (2) evidence suggesting that SMS text messaging is the most effective mHealth function for health behavior change, and (3) findings from focus groups and interviews indicating that Aboriginal and Torres Strait Islander women were high users of Facebook and SMS text messaging. As suggested in a recent pilot study of a smartphone app with Aboriginal Australians, a *one app fits all* approach is unlikely to be successful [24]. Using mHealth modalities commonly used by the target group to deliver a health intervention may appeal to more families.

### Strengths and Limitations

The first limitation of this research is that it was initiated by a research institution rather than by the community itself. True co-design should begin with completing a needs assessment with communities to see what the health priorities and potential solutions are for that community [38]. This is well described in a New Zealand co-design study [29,40]. To ensure that adequate time and resources are available for relationship building and needs assessment, both should be specified in protocols and funding applications so that sufficient budgets and time frames are allocated. Second, although the intervention covers a range of topics in brief, it does not cover any topic in depth. Although an mHealth intervention with wide-ranging topics seems to be preferred by participants, this may dilute the impact of the intervention on any one risk behavior. Providing links within the *Growin’ Up Healthy Jarjums* intervention to specific mHealth interventions for target behaviors may overcome this limitation by providing tips for more intense behavior change for those people who are *ready* to change. Third, because the participants were from only 3 NSW communities, the intervention may have limited generalizability in other Aboriginal and Torres Strait Islander communities. Aboriginal and Torres Strait Islander communities are made up of >250 language groups in which there is great diversity. If this intervention is to expand to other communities, systematic adaptation of the intervention would need to be carried out to ensure that the intervention is suitable to the context of each community [41].

A key strength of this study is that Aboriginal researchers (BH, NS, and BL) led engagement with participants and community organizations. Understanding the importance of trusted and strong cultural relationships, we only engaged with communities that the Aboriginal researchers had a relationship with, which likely resulted in trust as well as interest in participating in this study. Another strength of this study is the thorough reporting of the co-design processes. Inadequate reporting of intervention development was identified as a weakness in a recent systematic review on mHealth development (33). An additional strength is the involvement of primary health services and professionals. A recent review on health promotion programs in Aboriginal communities highlighted that an important consideration is to partner with primary health care services because they are well placed with frequent patient contact, health expertise, and often intricate knowledge of the community [42]. A final and important strength is that we developed a flexible portal for ongoing development and enhancement. The COVID-19 experience has reinforced how important it is to have alternatives to face-to-face health care. Useful additions in future iterations of this mHealth intervention might include development of a flexible platform suitable for inclusion of initiatives inspired by the COVID-19 pandemic, such as subsidized telehealth and videoconferencing. There are also opportunities to develop content on this platform in Aboriginal and Torres Strait Islander languages to better suit users.

### Comparison With Prior Work

Design characteristics identified in this study, including *social connection* and *family centeredness*, reflect Aboriginal and Torres Strait Islander perspectives of health. Connection to family, community, and culture, among other factors, are understood to be equal contributors to health [43]. Arabena et al [44] suggest that community and social connection can ultimately be the health promotion intervention for Aboriginal and Torres Strait Islander communities.

The finding that Aboriginal and Torres Strait Islander women were high users of social media, in particular Facebook, was unsurprising. Aboriginal and Torres Strait Islander health organizations have capitalized on the popularity of Facebook among Aboriginal and Torres Strait Islander people and have been early and adept users of social media for health promotion [25]. An Aboriginal-led social marketing campaign for health promotion, *Deadly Choices*, has 94,035 Facebook followers, 19,300 Instagram followers, and 9000 TikTok Followers [26,45].

As stated earlier, the methodologies used in this study were based on a co-design study for a health app with Māori and Pacific Islander people [29,40]. There were a number of similar co-design findings. In both studies, participants expressed a holistic view of health and connections to people and place as being central components of health. Participants in both studies talked about a family approach to health, rather than an individual approach, as well as accessible healthy activities in the community. Social support was found to be an important strategy in both studies.

Culture was also identified as important in both studies, although cultural representation may have been a more nuanced finding in the New Zealand study. In our Australian-based study, participants expressed the importance of Aboriginal and Torres Strait Islander representation in terms of designs, colors, images, people, organizations, and safety. Participants in the New Zealand study [29,40] expressed the need to include Māori knowledge, Whakapono (faith and spirituality), and Whakataukī (traditional proverbs), which were to be woven throughout the intervention; for example, the app depicts the completion of challenges as colored footsteps, which is analogous to the journey that the participants’ tūpuna (ancestors) embarked on. There may be differences in participants’ connection to culture. In Australia, up to 1 in 3 Aboriginal and Torres Strait Islander children were removed from their families during the period from the mid-1800s to the 1970s. These children are known as the Stolen Generations [39]. Of the 31 mothers in this study, 6 (19%) reported that they had family members from the Stolen Generations, whereas 13 (42%) were unsure. The effect of the Stolen Generations on the loss of culture is profound [39] and is likely reflected in the findings of this study. This intervention may, in a small way, help to promote culture through links to Aboriginal and Torres Strait Islander organizations, connection to mothers of Aboriginal and Torres Strait Islander children, and culturally safe health information.

### Conclusions

An mHealth intervention that included app, SMS text messaging, and Facebook page modalities was developed based on co-design findings. The intervention incorporates health behavior change theory, evidence-based information, and the preferences of Aboriginal and Torres Strait Islander women and health professionals. The next step of this research is to assess the acceptability and feasibility of the intervention in a pilot study. The pilot study will be conducted with the Aboriginal Health Services and NSW Health sites that participated in this co-design study. Participating mothers will also be invited to participate in the pilot study. If the *Growin’ Up Healthy Jarjums* intervention is shown to have adequate acceptability and feasibility, the next phase will be to measure its effectiveness in improving health knowledge and changing health behaviors. Assessing the effectiveness of this intervention will provide valuable evidence for the use of mHealth in improving the health and well-being of Aboriginal and Torres Strait Islander populations and contribute to the evidence for using co-design methodologies, both of which have been highlighted as gaps in the literature [46].

## Appendix

Multimedia Appendix 1 Behavior change techniques in SMS text messages.

## Abbreviations

AMS: Aboriginal Medical Service
mHealth: mobile health
NSW: New South Wales

## References

[1] National Aboriginal and Torres Strait Islander Health Plan 2013–2023. Australian Government Department of Health.

[2] Indigenous life expectancy and deaths 2019. Australian Institute for Health and Welfare.

[3] (2018). National Aboriginal and Torres Strait Islander health survey 2018-19. Australian Bureau of Statistics.

[4] (2020). 1.20 Infant and child mortality. Australian Institute of Health and Welfare: National Indigenous Australians Agency.

[5] Profile of Indigenous Australians 2020. Australian Government: Australian Institute for Health and Welfare.

[6] Australian Government: Australian Institute for Health and Welfare.

[7] Rennie E, Thomas J, Wilson C (2019). Aboriginal and Torres Strait Islander people and digital inclusion: what is the evidence and where is it?. Commun Res Pract.

[8] Thomas J, Barraket J, Wilson CK, Holcombe-James I, Kennedy J, Rennie E, Ewing S, MacDonald T, Royal Melbourne Institute of Technology, Telstra Corporation, Roy Morgan Research Centre Measuring Australia's digital divide: The Australian Digital Inclusion Index 2020. RMIT University, Centre for Social Impact, Telstra.

[9] Hall CS, Fottrell E, Wilkinson S, Byass P (2014). Assessing the impact of mHealth interventions in low- and middle-income countries--what has been shown to work?. Glob Health Action.

[10] Be He@lthy, Be Mobile (BHBM). World Health Organisation.

[11] (2019). Be Healthy, Be Mobile Annual Report 2018.

[12] Media usage amongst Aboriginal and Torres Strait Islander People (infographic). McNair Yellow Squares.

[13] Kirkham R, MacKay D, Barzi F, Whitbread C, Kirkwood M, Graham S, Van Dokkum P, McIntyre HD, Shaw JE, Brown A, O'Dea K, Connors C, Oats J, Zimmet P, Boyle J, Maple-Brown L (2019). Improving postpartum screening after diabetes in pregnancy: results of a pilot study in remote Australia. Aust N Z J Obstet Gynaecol.

[14] Phillips JH, Wigger C, Beissbarth J, McCallum GB, Leach A, Morris PS (2014). Can mobile phone multimedia messages and text messages improve clinic attendance for Aboriginal children with chronic otitis media? A randomised controlled trial. J Paediatr Child Health.

[15] Fletcher R, Hammond C, Faulkner D, Turner N, Shipley L, Read D, Gwynn J (2017). Stayin' on Track: the feasibility of developing Internet and mobile phone-based resources to support young Aboriginal fathers. Aust J Prim Health.

[16] Dobson R, Whittaker R, Bartley H, Connor A, Chen R, Ross M, McCool J (2017). Development of a culturally tailored text message maternal health program: TextMATCH. JMIR Mhealth Uhealth.

[17] Broom MA, Ladley AS, Rhyne EA, Halloran DR (2015). Feasibility and perception of using text messages as an adjunct therapy for low-income, minority mothers with postpartum depression. JMIR Ment Health.

[18] Marcolino MS, Oliveira JA, D'Agostino M, Ribeiro AL, Alkmim MB, Novillo-Ortiz D (2018). The impact of mHealth interventions: systematic review of systematic reviews. JMIR Mhealth Uhealth.

[19] Whittaker R, McRobbie H, Bullen C, Rodgers A, Gu Y, Dobson R (2019). Mobile phone text messaging and app-based interventions for smoking cessation. Cochrane Database Syst Rev.

[20] Milne-Ives M, Lam C, De Cock C, Van Velthoven MH, Meinert E (2020). Mobile apps for health behavior change in physical activity, diet, drug and alcohol use, and mental health: systematic review. JMIR Mhealth Uhealth.

[21] Romeo A, Edney S, Plotnikoff R, Curtis R, Ryan J, Sanders I, Crozier A, Maher C (2019). Can smartphone apps increase physical activity? systematic review and meta-analysis. J Med Internet Res.

[22] Hobson GR, Caffery LJ, Neuhaus M, Langbecker DH (2019). Mobile health for first nations populations: systematic review. JMIR Mhealth Uhealth.

[23] Ni Mhurchu C, Te Morenga L, Tupai-Firestone R, Grey J, Jiang Y, Jull A, Whittaker R, Dobson R, Dalhousie S, Funaki T, Hughes E, Henry A, Lyndon-Tonga L, Pekepo C, Penetito-Hemara D, Tunks M, Verbiest M, Humphrey G, Schumacher J, Goodwin D (2019). A co-designed mHealth programme to support healthy lifestyles in Māori and Pasifika peoples in New Zealand (OL@-OR@): a cluster-randomised controlled trial. Lancet Digit Health.

[24] Peiris D, Wright L, News M, Rogers K, Redfern J, Chow C, Thomas D (2019). A smartphone app to assist smoking cessation among aboriginal Australians: findings from a pilot randomized controlled trial. JMIR Mhealth Uhealth.

[25] Sweet MA (2013). Social media: new links for Indigenous health. Med J Aust.

[26] McPhail-Bell K, Appo N, Haymes A, Bond C, Brough M, Fredericks B (2018). Deadly choices empowering indigenous Australians through social networking sites. Health Promot Int.

[27] Finlay S, Wenitong M (2020). Aboriginal community controlled health organisations are taking a leading role in COVID-19 health communication. Aust N Z J Public Health.

[28] Petkovic JD, Duench S, Trawin J, Dewidar O, Pardo Pardo J, Simeon R, DesMeules M, Gagnon D, Hatcher Roberts J, Hossain A, Pottie K, Rader T, Tugwell P, Yoganathan M, Presseau J, Welch V (2021). Behavioural interventions delivered through interactive social media for health behaviour change, health outcomes, and health equity in the adult population. Cochrane Database Syst Rev.

[29] Verbiest ME, Corrigan C, Dalhousie S, Firestone R, Funaki T, Goodwin D, Grey J, Henry A, Humphrey G, Jull A, Vano M, Pekepo C, Morenga LT, Whittaker R, Mhurchu CN (2019). Using codesign to develop a culturally tailored, behavior change mHealth intervention for indigenous and other priority communities: a case study in New Zealand. Transl Behav Med.

[30] Ethical guidelines: key principles (2020). Aboriginal Health and Medical Research Council of NSW.

[31] Bratteteig T, Bødker K, Dittrich Y, Holst MP, Simonsen J, Simonsen J, Robertson T (2012). Methods: Organising principlesgeneral guidelines for participatory design projects. International Handbook of Participatory Design.

[32] Hall KK, Chang AB, Anderson J, Arnold D, Goyal V, Dunbar M, Otim M, O'Grady KA (2017). The incidence and short-term outcomes of acute respiratory illness with cough in children from a socioeconomically disadvantaged urban community in Australia: a community-based prospective cohort study. Front Pediatr.

[33] Riley WT, Rivera DE, Atienza AA, Nilsen W, Allison SM, Mermelstein R (2011). Health behavior models in the age of mobile interventions: are our theories up to the task?. Transl Behav Med.

[34] Champion V, Skinner C (2008). The health belief model. Health Behavior and Health Education: Theory, Research, and Practice; 4th edition.

[35] Michie S, Richardson M, Johnston M, Abraham C, Francis J, Hardeman W, Eccles MP, Cane J, Wood CE (2013). The behavior change technique taxonomy (v1) of 93 hierarchically clustered techniques: building an international consensus for the reporting of behavior change interventions. Ann Behav Med.

[36] Michie S, Free C, West R (2012). Characterising the ‘Txt2Stop’ smoking cessation text messaging intervention in terms of behaviour change techniques. J Smok Cessat.

[37] Abroms LC, Whittaker R, Free C, Mendel Van Alstyne J, Schindler-Ruwisch JM (2015). Developing and pretesting a text messaging program for health behavior change: recommended steps. JMIR Mhealth Uhealth.

[38] Boyd H, McKernon S, Mullin B, Old A (2012). Improving healthcare through the use of co-design. N Z Med J.

[39] (2021). Who are the stolen generations?. Healing Foundation.

[40] Te Morenga L, Pekepo C, Corrigan C, Matoe L, Mules R, Goodwin D, Dymus J, Tunks M, Grey J, Humphrey G, Jull A, Whittaker R, Verbiest M, Firestone R, Ni Mhurchu C (2018). Co-designing an mHealth tool in the New Zealand Māori community with a “Kaupapa Māori” approach. AlterNative Int J Indigenous People.

[41] Moore G, Campbell M, Copeland L, Craig P, Movsisyan A, Hoddinott P, Littlecott H, O'Cathain A, Pfadenhauer L, Rehfuess E, Segrott J, Hawe P, Kee F, Couturiaux D, Hallingberg B, Evans R (2021). Adapting interventions to new contexts-the ADAPT guidance. BMJ.

[42] Canuto KJ, Aromataris E, Burgess T, Davy C, McKivett A, Schwartzkopff K, Canuto K, Tufanaru C, Lockwood C, Brown A (2021). A scoping review of Aboriginal and Torres Strait Islander health promotion programs focused on modifying chronic disease risk factors. Health Promot J Austr.

[43] Calma T, Dudgeon P, Bray A (2014). Aboriginal and Torres Strait Islander social and emotional wellbeing. Working Together: Aboriginal and Torres Strait Islander Mental Health and Wellbeing Principles and Practice.

[44] Arabena K, Rowley K, MacLean S (2014). Building evidence about effective health promotion in Aboriginal and Torres Strait Islander communities. Aust J Prim Health.

[45] Deadly numbers 2018. Deadly Choices.

[46] Eyles H, Jull A, Dobson R, Firestone R, Whittaker R, Te Morenga LT, Goodwin D, Mhurchu CN (2016). Co-design of mHealth delivered interventions: a systematic review to assess key methods and processes. Curr Nutr Rep.

