# Supplementary material for: Development of a Maternal and Child mHealth Intervention With Aboriginal and Torres Strait Islander Mothers: Co-design Approach
Source: JMIR Form Res. 2022 Jul 8;6(7):e33541. doi: 10.2196/33541 (PMC9308065; doi:10.2196/33541)
Supplement: Multimedia Appendix 1 [file formative_v6i7e33541_app1.docx]

**Multimedia Appendix 1**

**BCT clusters and components coded in text messages (112 messages)**

| **Behaviour change cluster** | **Component** | **N (100)** |
| --- | --- | --- |
| 9. Comparison of outcomes | 9.1 Credible source | 55 (28.6) |
| 5. Natural consequences | 5.1 Info about health consequences. | 36 (18.6) |
| 1. Goals and planning | 1.4 Action planning | 25 (13.0) |
| 4. Shaping knowledge | 4.1 Instruction on how to perform a behaviour | 17 (89) |
| 10. Reward and threat | 10.4 social reward | 11 (5.7) |
| 3. Social support | 3.1 Social support (unspecified) | 8 (4.2) |
| 5. Natural consequences | 5.6 Info about emotional consequences | 5 (2.6) |
| 13. Identity | 13.1 Identification of self as role model | 4 (2.0) |
| 7. Associations | 7.1 Prompt/cues | 4 (2.0) |
| 6. Comparison of behaviour | 6.2 Social comparison | 3 (1.6) |
| 8. Repetition and substitution | 8.7 Graded task | 3 (1.6) |
| 12. Antecedents | 12.6 Body changes | 3 (1.6) |
| 11. Regulation | 11.1 Pharmacological support | 3 (1.6) |
| 1. Goals and planning | 1.1 Goal setting (behaviour) | 2 (1.0) |
| 4. Shaping knowledge | 4.2 Information about antecedents | 2 (1.0) |
| 9. Comparison of outcomes | 9.2 Pros/cons | 1 (0.5) |
| 12. Antecedents | 12.3 Avoidance/reducing exposure to cues for the behaviour | 1 (0.5) |
| 10. Reward and threat | 10.9 Self-reward | 1 (0.5) |
| 7. Associations | 7.8 Associative learning | 1 (0.5) |
| 11. Regulation | 11.2 Reduce negative emotions | 1 (0.5) |
| 12. Antecedents | 12.4 Distractions | 1 (0.5) |
| 15. Self-belief | 15.3 Focus on past success | 1 (0.5) |
| 15. Self-belief | 15.1 Verbal persuasion about capability | 1 (0.5) |
| **15** | **23** | **192 (100)** |
